# Supplementary material for: The interactome of intact mitochondria by cross-linking mass spectrometry provides evidence for coexisting respiratory supercomplexes
Source: Mol Cell Proteomics. 2017 Dec 8;17(2):216–32. doi: 10.1074/mcp.RA117.000470 (PMC5795388; doi:10.1074/mcp.RA117.000470)
Supplement: Supplemental Data [file supp_RA117.000470_133922_0_supp_23978_fzffwf.zip › spectra_annotation/mito_spectra_annotation/394-1-4-1-11-1.pdf]

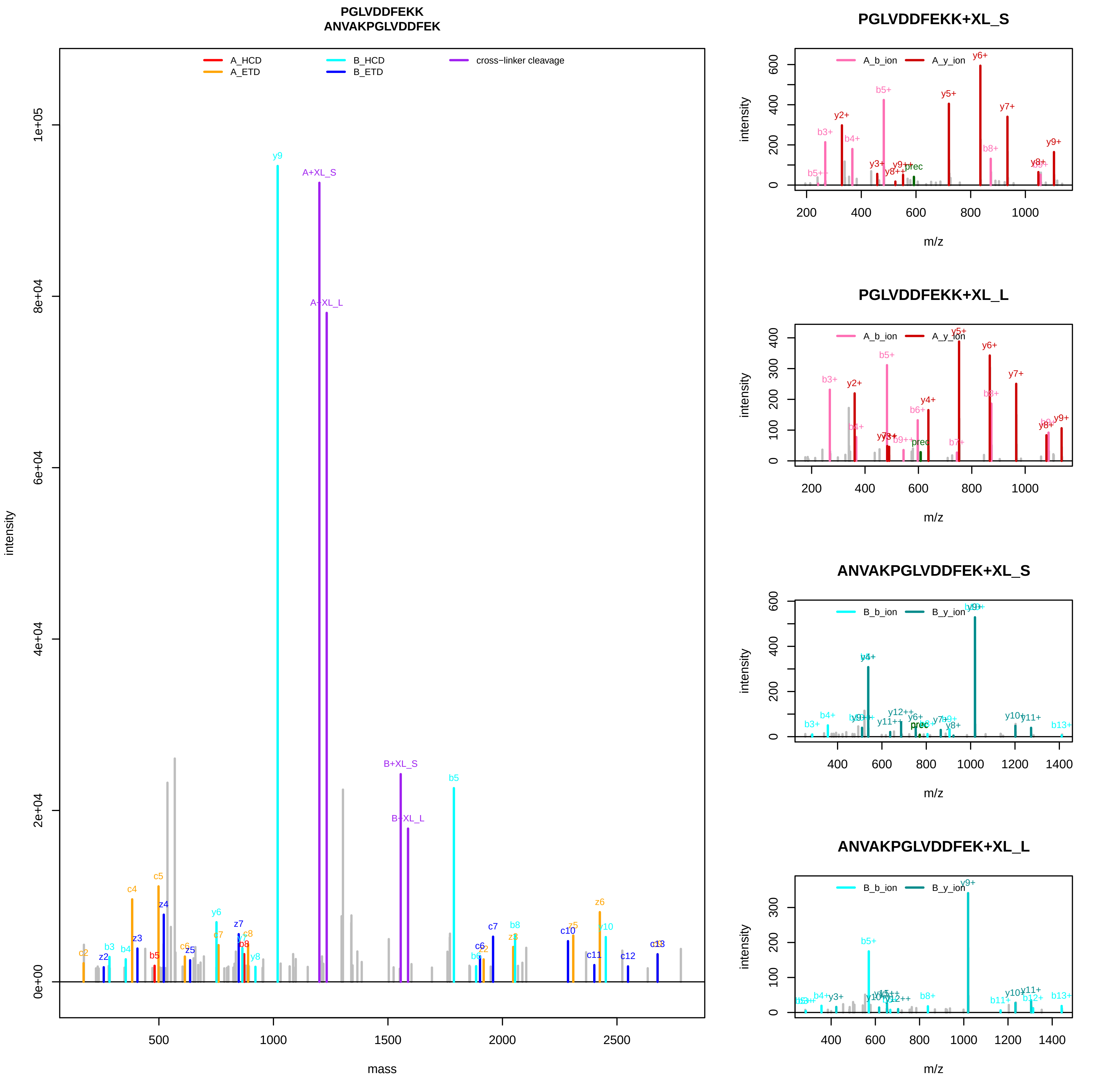

PGLVDDFEKK
ANVAKPGLVDDFEK
A_HCD
A_ETD
B_HCD
B_ETD
cross−linker cleavage
intensity
mass
0e+00
2e+04
4e+04
6e+04
8e+04
1e+05
500
1000
1500
2000
2500
y9
A+XL_S
A+XL_L
B+XL_S
B+XL_L
b5
c2
z2
b3
b4
z3
b5
c4
c5
z4
c6
z5
y6
c7
z7
y7
c8
b8
y8
c6
b6
z2
c7
b8
z3
c10
z5
c11
z6
y10
c12
c13
PGLVDDFEKK+XL_S
A_b_ion
A_y_ion
b5++
b3+
y2+
b4+
b5+
y3+
y9++
y8++
prec
y5+
y6+
b8+
y7+
y8+
y9+
intensity
m/z
PGLVDDFEKK+XL_L
b3+
y2+
b4+
b5+
y3+
b9++
b6+
prec
y4+
y5+
b7+
y6+
b8+
y7+
y8+
y9+
intensity
m/z
ANVAKPGLVDDFEK+XL_S
B_b_ion
B_y_ion
b3+
b4+
y4+
y12++
y11++
y6+
prec
y7+
y8+
y9+
y10+
y11+
b13+
intensity
m/z
ANVAKPGLVDDFEK+XL_L
B_b_ion
B_y_ion
b3+
b4+
y3+
b5+
y10++
y12++
b8+
y9+
b11+
y10+
y11+
b12+
b13+
intensity
m/z
